# IngridKG: A FAIR Knowledge Graph of Graffiti

**DOI:** 10.1038/s41597-023-02199-8

**Published:** 2023-05-25

**Authors:** Mohamed Ahmed Sherif, Ana Alexandra Morim da Silva, Svetlana Pestryakova, Abdullah Fathi Ahmed, Sven Niemann, Axel-Cyrille Ngonga Ngomo

**Affiliations:** 1 DICE Research Group, Department of Computer Science, Paderborn University, Paderborn, 33098 Germany; 2 Institute for German Language and Comparative Literature, Paderborn University, Paderborn, 33098 Germany

**Keywords:** Arts, Society

## Abstract

Graffiti is an urban phenomenon that is increasingly attracting the interest of the sciences. To the best of our knowledge, no suitable data corpora are available for systematic research until now. The Information System Graffiti in Germany project (Ingrid) closes this gap by dealing with graffiti image collections that have been made available to the project for public use. Within Ingrid, the graffiti images are collected, digitized and annotated. With this work, we aim to support the rapid access to a comprehensive data source on Ingrid targeted especially by researchers. In particular, we present IngridKG, an RDF knowledge graph of annotated graffiti, abides by the Linked Data and FAIR principles. We weekly update IngridKG by augmenting the new annotated graffiti to our knowledge graph. Our generation pipeline applies RDF data conversion, link discovery and data fusion approaches to the original data. The current version of IngridKG contains 460,640,154 triples and is linked to 3 other knowledge graphs by over 200,000 links. In our use case studies, we demonstrate the usefulness of our knowledge graph for different applications.

## Background & Summary

Graffiti is increasingly attracting the interest of different disciplines like linguistics, art history, anthropology and sociology. Until now, suitable data corpora for systematic research have been lacking. Depending on the research interests, further information about the place where a graffiti is painted, time when it is created, artists who created it, content and language are relevant in addition to the photographs themselves. Although countless photographs of graffiti can be found on the internet, they are not useful for research because the image rights often cannot be determined. Furthermore, the images are usually missing the qualitative metadata containing the aforementioned information about the graffiti.

The research project Ingrid (https://www.uni-paderborn.de/forschungsprojekte/ingrid/projekt) *“Information System Graffiti in Germany”* closes this gap. In the summer of 2012, the idea was born to establish an interdisciplinary graffiti database within the framework of an interdisciplinary cooperation between the art historians at the *Karlsruhe Institute of Technology* (KIT) and the *Paderborn University*. Since the practice of graffiti writing is often characterized by a mix of pictorial and written language, an interdisciplinary orientation (i.e., linguistics and art history) of the project was obvious.

Since 2016, Ingrid has been funded by the German research foundation DFG (https://www.dfg.de). The first project phase, from 01.04.2016 to 30.06.2019, aimed at building a terminology to systematically document and analyze graffiti in Germany. The categories within the graffiti terminologies are created based on our analysis of the available graffiti. The creation of our graffiti terminology has been an ongoing process which is reviewed constantly. Within the first phase of the project, over 40,000 graffiti from the *police department of Mannheim* and the private collection of *Peter Kreuzer* from *Munich* were annotated and made accessible for research purposes.

In the current second project phase, from 01.07.2020 to 30.06.2023 (also funded by DFG), photos provided by the *police department in Munich* as well as photos from the private collector *Dirk Kreckel* among others have been annotated. Dirk Kreckel has been photographing graffiti at important hot spots in Germany like Hamburg, Wiesbaden and Dortmund for decades. The aim of the second phase of Ingrid is to test and use crowd-sourcing methods for collecting and annotating the graffiti images. Therefore, from 01.05.2022 to 30.06.2022, the citizens of the city of Paderborn had the opportunity to document photos of graffiti in the city area via the LingScape application (https://lingscape-app.uni.lu). The photos then were transferred to our database of Ingrid.

The Ingrid database currently consists of approximately 136,000 photos from Mannheim, Munich, Cologne, Berlin, Leipzig, Hamm, Dortmund, Münster and Paderborn from the years 1983 to 2019. The images of the graffiti are collected from archives, private property and police inventory. A continuous expansion of the graffiti sources is planned within the project. By building this extensive graffiti database, Ingrid is preserving ephemeral art by documenting, digitizing and analyzing graffiti in the public space. Based on the extensive, secure and high-quality research data within the Ingrid, the developments and changes in the phenomenon of graffiti can be explored over longer periods of time. Ingrid enable the researchers to investigate the visual aesthetics, specific scripturality, notational iconicity, urban location, social function and meaning of each graffiti. After the annotation of the content of the graffiti images, the resulted data is transferred to a permanent domain that provide interfaces and connections to existing standards such as the *Integrated Authority File* (GND). Through connections to the major meta-databases and data infrastructure programs of the participating subjects, such as Prometheus (https://prometheus.io) and CLARIN-D (https://www.clarin-d.net/de/), an interdisciplinary networking is achieved. As of September 2022, 87,909 annotated graffiti are currently accessible for the public use from the project web site. For accessing our graffiti database, users need to log in to (https://media.uni-paderborn.de/). The original images of the graffiti may be used publicly under the license Attribution-ShareAlike 4.0 International (CC BY-SA 4.0), (https://creativecommons.org/licenses/by-sa/4.0/). On the other hand, our generated metadata are available under the CC0 license (https://creativecommons.org/share-your-work/public-domain/cc0/). Our knowledge graph, IngridKG, is publicly available under the Creative Commons Attribution 4.0 International (CC BY 4.0) license. See (https://creativecommons.org/licenses/by/4.0/) for more details. By default, the provided images contain a watermark, but we can provide the images in higher resolution and without a watermark on demand in response for individual requests.

In this paper, we present IngridKG, a comprehensive RDF knowledge graph of annotated graffiti images. Our knowledge graph follows the Linked Data lifecycle^1^. We provide detailed representation of the annotated graffiti in RDF including properties like graffiti’s text, location, creators and annotators. Resources such as graffiti and sprayer crews augment the original data and make it easier to process for the sake of question answering and machine learning.

**Fig. 1:**
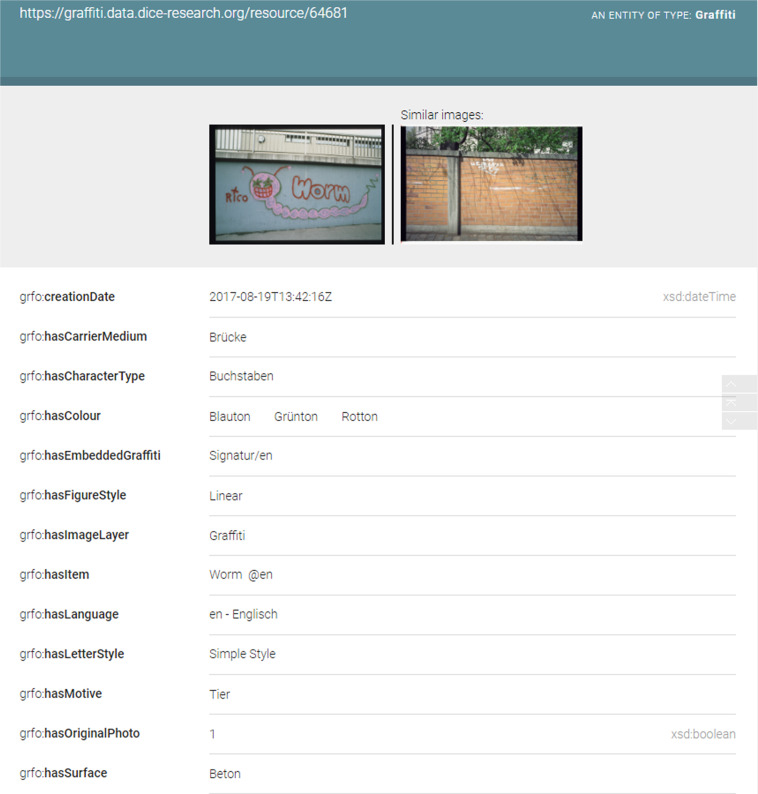
Screenshot of the resource page grfr:64681.

Our knowledge graph abides by the *FAIR principles*^2^: It is **Findable** by virtue of being annotated with rich metadata and indexable by search engines. We make it **Accessible** by providing our data via an RDF dump download (10.5281/zenodo.7560242) as well as (https://hobbitdata.informatik.uni-leipzig.de/INGRID/). All resources within the knowledge graph are dereferenceable via HTTP IRIs, which can be accessed via LodView (https://lodview.it/) or via the IngridKG’s SPARQL endpoint (https://graffiti.data.dice-research.org/sparql). For instance, Fig. 1 shows the LodView’s visualization of the resource (grfr:64681). We also make our knowledge graph **Interoperable** by employing standard vocabularies, e.g., for crews, crew members and annotators, as well as through the links to 3 knowledge graphs including DBpedia, WikiData and LinkedGeoData. Finally, we make IngridKG **Reusable** by associating the data with clear provenance and licensing information as well as by reusing popular vocabularies such as schema and rdfs ourselves.

IngridKG allows to obtain a deeper analysis of textual data that cannot be carried out with the user search in our database. For example, a SPARQL query can show which letters are most often used in sprayer names. The answer to this question is important because the letters in a sprayer or crew name in a graffiti writing are based on principles of visual design. A graffiti writing can be described as a stylized signature on the wall that is based on letters, local environment and stylistic elements. We could measure the degree of complexity of a graffiti by computing the number of letters, the use of different colors, or the number of style elements. These correlations between subcultural community, public space, time, quantity and quality are important questions for empirical studies in different disciplines of science such as art history, onomastics, linguistics, or social science. Other potential *use cases* of our knowledge graph include:

- Finding all graffiti painted by a certain crew and showing relationships of a social practice
- Analyzing local subcultural practices by researching the style in a certain city
- Investigating language economy and the complexity of written language
- Focusing on language and environment by linking information about the city

There are a number of other projects focused on the graffiti data collection and annotation. For instance, the project SprayCity (https://spraycity.at/) archives a digital collection regarding graffiti in Austria. The Indigo project (https://projectindigo.eu/) aims to provide the groundwork for thoroughly documenting, disseminating and analyzing the over 13 km of unbroken graffiti along Vienna’s *Donaukanal* (English: Danube Canal) in the next ten years. Finally, the Storm project (https://www.heritageresearch-hub.eu/project/storm/) aims at safeguarding of cultural heritage through technical and organisational resource management.

## Methods

### Graffiti annotation

The Ingrid database is hosed by the EasyDB. The database contains over 130,000 photographs of graffiti. The images were provided to Ingrid by different sources. A large part of the pictures comes from the police departments in Mannheim, Cologne and Munich. Another part consists of photographs from private collections and public archives. In order to be able to use the images, a contract is made between the Paderborn University and the copyright holders, which transfers the rights for public use of the images to the Ingrid project. This agreement allows Ingrid users to publish images as well as images’ metadata publicly (licensing: (CC BY-SA 4.0) for the original images, CC0 for our generated metadata and (CC BY 4.0) for IngridKG). In addition to the collection of images, a central task of the Ingrid project is to develop standards for research and analysis of graffiti. The development of these standards led to an ontology that captures constitutive aspects of graffiti (i.e., imagery, scripturality, context, locality, materiality, time, actors, etc.). The ontology also takes into account categories that are relevant to the graffiti scene itself. These include the distinction of certain letter styles (e.g., simple style, wild style and bubble style), the typology (e.g., pieces, tags and characters), and the use of typical stylistic elements (e.g., crowns, arrows and outline). This strategy led to an ontology based on folk categories as well as linguistic categories or art historical terms. The Information about the location, the recording date and the origin of the images is automatically imported by the *Information and Media Technologies Centre* (IMT) (https://imt.uni-paderborn.de/en/) of Paderborn University during the import process from the original mediums such as DVD, CD and memory sticks. When imported into the database, each image is assigned a systemID, which allows users to uniquely cite images. The systemID allows other system users to track and repeat the analyses. The annotation is done by six annotators in two different teams: one in Paderborn (linguistics) and the other in Karlsruhe (art history). The team in Paderborn focuses on aspects of scripturality such as the analysis of language, grammar and semiotics. The team in Karlsruhe focuses on aspects of imagery such as the shape of letters, the design of figurative elements, and the choice of colors.

### The annotation process details

The annotation process is carried out by selecting the proper categories and subcategories for each graffiti by the annotators. For example, the category *type* contains a number of different subcategories (e.g., *tag*, *stencil*, *piece*). Table 1 shows a sample of the annotations’ categories and its respective sub-categories. Please refer to our online annotation manual (https://dice-group.github.io/ingrid.github.io/) for the complete categories and subcategories table. An overview of the central categories are given in 2 In addition to the selection of fixed categories, our annotators are able also to fill out open text fields when annotating a graffiti (e.g., *item*, *text*, *remarks*) The category *text* is systematic representation of all graffiti in written form, which is very usable as graffiti is often difficult to read due to its deformation of letters. The open text field *remarks* gives useful information with some background knowledge about the topic or the motif that can help the users to understand the meaning of a graffiti. In order to keep the Inter-Annotator Agreement (IAA) high, the annotation on each individual category and each open text field is based on specific rules defined in an annotation manual. The annotation manual contains all names of the used categories and terms with the corresponding classification criteria. For example, the category *piece* is defined as a large, multicolored graffiti with areal letters. The central element of a piece is usually decorative with typical stylistic elements and can include figurative representations (characters). Smaller forms of graffiti are often embedded in a piece (e.g., tags, comments or dating). For more information a German and an English version of the complete manual is available on the project homepage and can be downloaded via the following links: German manual (https://www.uni-paderborn.de/fileadmin/ingrid/INGRID_Manual_Oktober_2019.pdf), English manual (https://github.com/dice-group/ingrid.github.io/blob/main/INGRID_Manual_Oktober_2019en.pdf). Even though the manual defines clear rules for annotation, in some cases graffitis appear that cannot always be clearly assigned to a category. In such cases, the annotation of a graffiti is clarified within our weekly meeting. Once the annotation of the images of the graffiti is completed, the images are made available for search in our instance of the EasyDB database (https://media.uni-paderborn.de/). Moreover, we provide a public full images dump via the Hobbit data server at https://hobbitdata.informatik.uni-leipzig.de/INGRID/ingrid_public_images.tar.gz.

**Table 1:** Annotation categories.

Categories	Subcategories
Technology	Pencil, Roller, Pen, Pressure scratch, chalk, Stencil, Spray can, Other
Carrier medium	Trailer, Vending machine, Railway line, Tree/Plant, Ground, Mailbox, Bridge, Bus, Container, Downpipe, Window/Blind, Railing/Handrail, Closed interior, Freight train, Hall of Fame, Stop noise barrier, Truck, Mast, Waste/Disposal container, Park bench, Passenger train, Car, Column/Pillar/Support, Display, Case Sign, Play/Sports equipment, Sticker, Road salt container, Electricity/Distribution box, Telephone box, Gate, Stairs, Door, Subway/S-Bahn Subway/S-Bahn/Passenger train - inside underpass, Wall Fence, Other, Undefined
Surface	Asphalt, Concrete, Glass, Wood, Ceramics, Plastic, Metal, Natural stone, Paper/Cardboard, Plaster, Exposed, Aggregate, Concrete Brick, Other, Undefined
Type	Character, Comment, Co-Construction, Composition, Piece/Writing/Style, Sketch, Saying/Concept call, Stencil, Tag(s), Throw Up, Otherwise
Subject area	Drugs, Erotic, Film, Peace, Football, Sports, Violence, Culture Art, Love, Music, Politics, Police, Racism, Religion, School, Death, Environment, Behavioural standard, Economy, Other, None

### Knowledge graph generation

We start the process of our knowledge graph generation by creating our Ingrid ontology. In particular, we define the basic classes (i.e., graffiti, annotator, crew and crew members) and predicates associated with each of the class’s entities. We detail our ontology generation process in the next section. Based on our ontology, we developed *Python* scripts for converting the database records of the graffiti images into our RDF IngridKG. We then link our created IngridKG with external knowledge graphs. Currently, we link IngridKG to the knowledge graphs of LinkedGeoData, WikiData and DBpedia. We details our linking process in the *Linking Section*.

## Data Records

Our knowledge graph, IngridKG, is available at the open science portal Zenodo^3^. Currently, Zenodo’s repository mirrors the data present in our SPARQL endpoint (https://graffiti.data.dice-research.org/sparql/) and in our data dump (https://hobbitdata.informatik.uni-leipzig.de/INGRID). in particular, we include the public version of IngridKG with graffiti resources and respective linking information, the ontology file, the dataset metadata expressed with the standardized VoID vocabulary, the similarity scores between the different graffiti resources in the form of reified statements, and the images of the publicly available graffiti. Furthermore, we provide a README file with a short description of each file within the repository. In Table 2, we list the files available at our Zenodo^3^ together with its respective descriptions. In this section, we begin by describing the **structure** of our ontology, where we introduce each of its classes, name spaces and data model. We then introduce our knowledge graph linking procedures. Finally, we detail how we automate our knowledge graph generation process.

**Table 2:** List of files at our Zenodo repository.

File name	Description
public_rdfGraffiti.ttl	Contains the public version of IngridKG, including the graffiti resources and the linking information.
ontology_v3.ttl	Contains the ontology of INGRID’s KG.
void.ttl	Contains metadata on the dataset
ingrid_similar_images_public_1.ttl	First part of the similarity scores file.
ingrid_similar_images_public_2.ttl	Second part of the similarity scores file.
ingrid_similar_images_public_3.ttl	Third part of the similarity scores file.
ingrid_public_images.tar.gz	Compressed file with all the public images of the graffiti, where the name of each image file reflects the graffiti ID in the KG.

Our knowledge graph creation process is implemented in *Python* 3.6 with *RDFLib* 5.0.0 (https://github.com/RDFLib/rdflib). We make our source code publicly available (https://github.com/dice-group/Ingrid) to ensure the *reproducibility* of our results and the rapid conversion of novel graffiti database versions. We present some statistics regarding the increasing size of the IngridKG’s resources over time in Table 4.

**Table 3:** Technical details of IngridKG.

Name	IngridKG
**Zenodo archive**	10.5281/zenodo.7560242
**IngridKG license**	Creative Commons Attribution 4.0 International (https://creativecommons.org/licenses/by/4.0/)
**Example resource**	https://graffiti.data.dice-research.org/resource/64681
**IngridKG dump**	https://hobbitdata.informatik.uni-leipzig.de/INGRID/
**Archived dump**	https://hobbitdata.informatik.uni-leipzig.de/INGRID/archive
**Sparql endpoint**	https://graffiti.data.dice-research.org/sparql/
**IngridKG graph**	https://hobbitdata.informatik.uni-leipzig.de/INGRID/public_rdfGraffiti.ttl
**Void file**	https://hobbitdata.informatik.uni-leipzig.de/INGRID/void.ttl
**Version date**	July 5, 2022
**Version Number**	4.0
**Ontology**	https://hobbitdata.informatik.uni-leipzig.de/INGRID/ontology_v3.ttl
	10.5281/zenodo.7560242
	https://github.com/dice-group/ingrid.github.io/blob/main/ontology_v3.ttl
Source code	https://github.com/dice-group/Ingrid
Software license	GPL 3.0 (https://www.gnu.org/licenses/gpl-3.0
Graffiti images archive	10.5281/zenodo.7759189
	https://hobbitdata.informatik.uni-leipzig.de/INGRID/ingrid_public_images.tar.gz
Graffiti images license	CC BY-SA 4.0, (https://creativecommons.org/licenses/by-sa/4.0/)
Database	https://media.uni-paderborn.de/
Annotation manuals	https://dice-group.github.io/ingrid.github.io/
Annotations license	CC0, (https://creativecommons.org/share-your-work/public-domain/cc0/)

**Table 4:** IngridKG statistics.

	29.06.2021	30.08.2021	28.02.2022	05.07.2022	Latest
Distinct number of all resources	640,536	803,675	835,597	841,167	840,420
Distinct number of graffiti	100,758	125,962	130,692	130,831	130,689
Distinct number of sprayer crews	15,044	16,656	21,616	23,380	23,272
Distinct number of graffiti symbols	0	0	0	46	46
Distinct number of crews	348	348	348	0	0
Distinct number of crew members	581	581	581	948	948
Distinct number of cities	149	160	165	178	178
Distinct number of image files	523,242	659,470	681,378	683,957	682,748
Distinct number of collections	0	0	0	451	451

### Ontology design

The ontology behind our knowledge graph is derived from the source from which it was extracted, i.e., the database of the graffiti image annotations. The ontology is designed to enable search, question answering and machine learning.

**Fig. 2:**
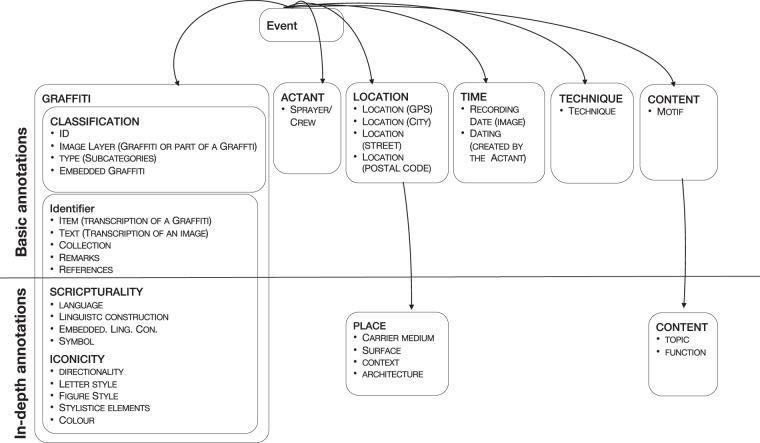
Annotations categories.

As part of our continuous **ontology refinement** work, we fused the instances of the Crew class into the SprayerCrew class, which is the reason of the disappearance of the Crew class instances and the increased number of the instances of the SprayerCrew class starting from the 05.07.22 version. Moreover, we added the Collection class in the same version Fig. 2.

#### RDF namespaces

**Fig. 3:**
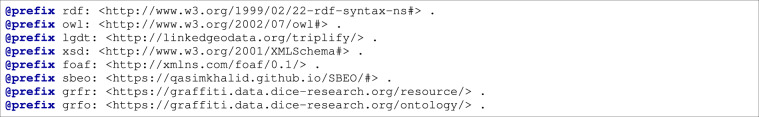
List of all used vocabularies in IngridKG.

To facilitate the reusability of our knowledge graph, we represent our data in widely used vocabularies and namespaces as shown in Fig. 3.

#### RDF data model

**Fig. 4:**
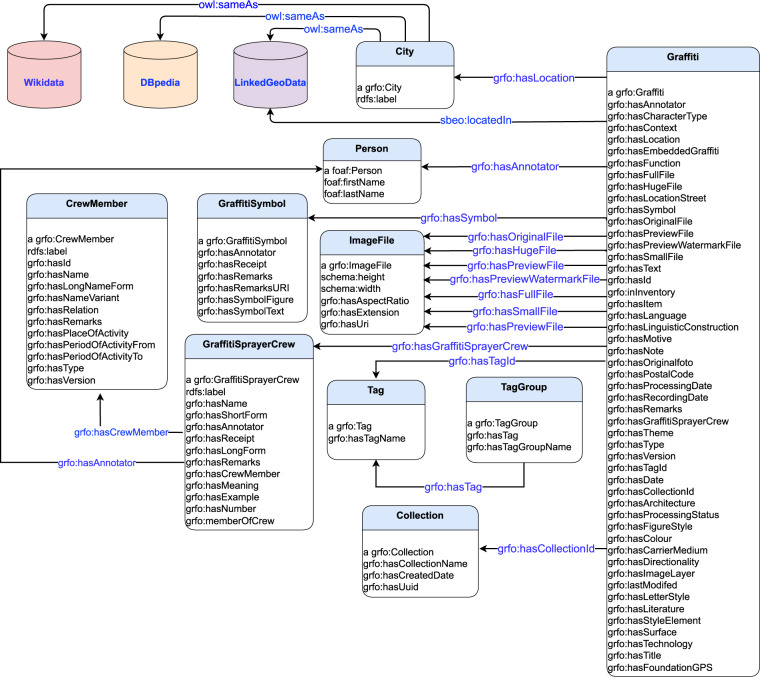
UML class diagram for the IngridKG ontology.

Figure 4 shows important classes (e.g., graffiti, crew, crew member, person, image file and city) as well as predicates (e.g., graffiti’s location, annotator and text within a graffiti).

##### Graffiti

**Fig. 5:**
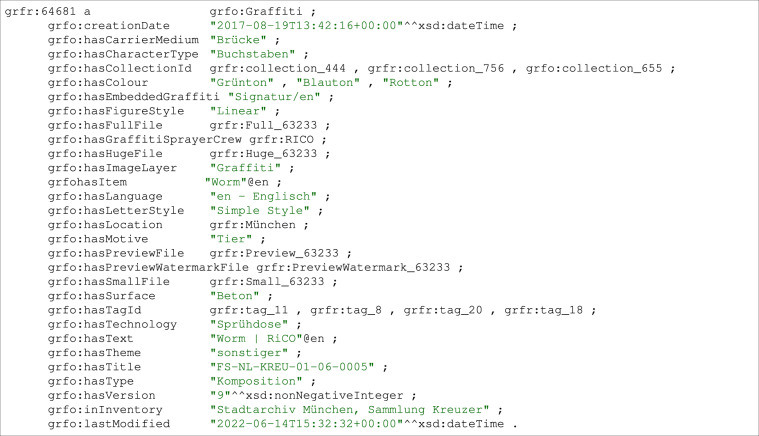
Example graffiti resource.

We represent graffiti as instance of class grfo:Graffiti. Each graffiti instance contains information regarding the graffiti’s provenance, contents, creators and annotators. Important attributes regarding graffiti contents include text, character type, language, color, theme, text direction, carrier medium and context. For each graffiti, we store **provenance information**. In particular, using the predicate grfo:inInventory, we explicitly state the original inventory where the raw image file of the graffiti come from. IngridKG also allows referencing to the original image raw files as well as the place and time where/when the image is taken. Moreover, we store the time when we annotate the resource. The URIs of our generated Graffiti resources follow the format https://graffiti.data.dice-research.org/resource/graffitiId where graffitiId is the unique id for each graffiti within IngridKG. For example, the original image of the graffiti resource presented in Fig. 5 is created on 2017-08-19 at 13:42:16 o’clock from the inventory of Stadtarchiv Munchen, Sammlung Kreuzer and the last annotation work of the image is on 2022-06 at 14:00 to 15:32:327 o’clock.

##### Collection

Each graffiti in IngridKG is linked to the collection it came from. There are five different types of collections: (i) Police Department Mannheim, (ii) Police Department Munich, (iii) Police Department Cologne, (iv) The collection of Dirk Kreckel and (v) The collection of Peter Kreuzer.

##### Annotator

Our annotators are represented in the FOAF (http://xmlns.com/foaf/spec/http://xmlns.com/foaf/spec/) vocabulary. In particular, we include each annotator’s first, middle and last name as well as mail address and institute. Each graffiti instance is linked to its respective annotators via the grfo:hasAnnotator predicate (as shown in Fig. 4). For data protection reasons, we can not provide examples of the annotator resources. *(Note that, that is the only part of our knowledge graph that is not publicly available due to data protection reasons.)*

##### Crew

**Fig. 6:**
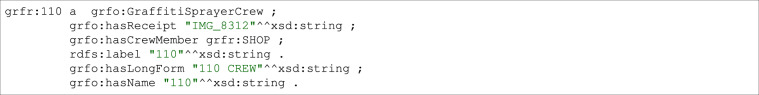
Example of a crew representation.

Each graffiti is linked to the crews that painted it via the grfo:hasGraffitiSprayerCrew predicates. Each crew instance include information related to crew name in short and long form, members and any spacial notes. Each crew is also linked to its annotator via the grfo:hasAnnotator predicate. In Fig. 6, we provide an example of a crew resource.

##### Crew members

**Fig. 7:**
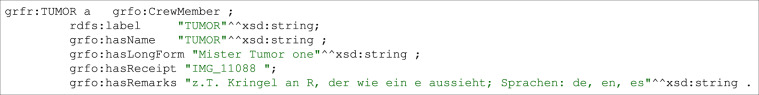
Example of a crew member.

Each crew contains one or more crew members. Therefore, we store crew members’ corresponding information as instance of class grfo:CrewMember. In particular, we store for each crew member his/her name, name variants, abbreviations and remarks. See Fig. 7 for an example of a crow member resource.

##### Image files.

For each graffiti, we store its original image files in various qualities and sizes. Each grfo:ImageFile instance include the image file’s height, width, aspect ratio, extension and URI.

### Linking

We link our dataset to other data sources to ensure its reusability and integrability as well as to improve its use for search, question answering and structured machine learning. We generate links from our graffiti resources to publicly available related knowledge bases. In our linking process, we rely on Limes^4^ as it is a state-of-the-art declarative link discovery framework with open source implementation that can be easily adopted and configured. We manually created a Limes configuration file for each linking task. All Limes configuration tasks are available from the project web site (https://github.com/dice-group/Ingrid).

#### Linking graffiti

We link IngridKG’s resources of type grfo:Graffiti to LinkedGeoData resources of type lgdo:Building. In particular, we configure Limes to declare a link (i.e., sbeo:locatedIn) if both city name and street name of the grfo:Graffiti and the lgdo:Building are matched using the *jaccard* similarity of at least 90%. Following the same method, we also link our resources of type grfo:Graffiti to LinkedGeoData resources of type lgdo:HighwayThing.

#### Linking cities

Each graffiti resource in our dataset (i.e., of type grfo:Graffiti) is linked to a city resource (i.e., of type grfo:City) using the predicate sbeo:locatedIn as shown in Fig. 4. We link grfo:City resources from IngridKG to the LinkedGeoData knowledge graph using the owl:sameAs predicates. In particular, we link resources of type grfo:City from our knowledge graph to resources of type lgdo:City from LinkedGeoData dataset. In particular, we configure Limes to declare a match once the *levenshtein* similarity among the rdfs:label of a grfo:City resource from IngridKG and a lgdo:City resource from LinkedGeoData is above 90%. In a similar way, we link the IngridKG’s resources of a grfo:City to DBpedia resources of type dbo:Location. We configured Limes to declare a link once the *jaccard* similarity between the rdfs:label of grfo:City resource and a dbo:Location resource is above 60%. Finally, we linked our grfo:City resources to the ones from WikiData of type wd:Q183. For instance, Limes discovered 208,942 sbeo:locatedIn links between IngridKG and LinkedGeoData, and 65 owl:sameAs links between IngridKG and WikiData.

#### Linking postal codes

We link each of the graffiti with known postal code with LinkedGeoData locations with the exact match lgdo:postalCode via the predicate sbeo:locatedIn. As the graffiti are located in Germany and postal codes are not unique worldwide, we configured Limes to restrict the postal codes to the ones in Germany (i.e., postal codes which are managed by the *Deutsche Post*).

#### Linking exact duplicated and near duplicated Images

**Fig. 8:**
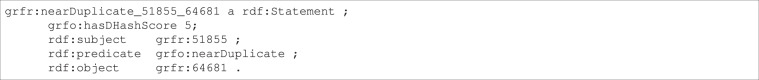
Example of near duplicate resources of grfr:64681.

We linked each graffiti resource in our Knowledge graph to all its image’s duplicates and near duplicates. Near duplicate images of graffiti generally exists as a result of one graffiti being captured from different view points, distance, illumination conditions of different resolution. In particular, we used the predicate grfo:nearDuplicate to link each graffiti resource to its exact duplicated and near duplicated resources. For example, we can see Fig. 8 that the graffiti resource grfr:64681 has the near duplicates resources grfr:51855. Technically, we first used the Python package imagededup^5^ to encode all the images. We then used image duplication technique DHash (https://github.com/idealo/imagededup) to carry out our near duplication experiments.

## Technical Validation

### Annotation validation

The annotators use a manual in which the rules for the annotation are defined. Cases of uncertainty are discussed in a weekly team meeting in Zoom. In addition, the annotators use the platform Slack to exclude any uncertainty. Before the annotation starts, it is checked whether a comparable case has already been annotated in order to keep the inter-annotator agreement as high as possible.

### Ontology validation

We build our knowledge graph IngridKG based on our defined ontology via the usage of our knowledge graph automatic generation scripts (https://github.com/dice-group/Ingrid). In particular, we make sure that each of our knowledge graph conversion scripts respect all the defined ontology constrains within our ontology. For instance, all our generation scripts always creates the relation grfo:creationDate with the domain of grfo:Graffiti and the range of xsd:dateTime. Therefore, we do not need to run any ontology validation of our knowledge graph after it is created.

### Linking validation

As mentioned in the linking section, we use the link discovery framework Limes for generating the links among Ingrid and the external knowledge graphs of DBpedia, LinkedGeoData and WikiData. To validate the automatically generated links by Limes, we ran a manual annotation of the generated links by at least three annotators. In particular, each annotator manually check the correctness of each link as being True of False. Afterwords, we gather the common voting of the annotator as the mutual agreement for each link. We then remove all links with a False mutual agreement, if any. Finally, we add the True mutually agreed linked to our knowledge graph. In Table 5, we provides an example of the result of manual annotation of city resources from Ingrid and WikiData, where each like is manually checked for correctness by three annotators and the mutual agreement is presented in the last column. Overall, our manual annotators define less than 5% of the links generated by Limes as wrong and all of them were removed from IngridKG.

**Table 5:** Manual annotation results of links generated by Limes among cities of IngridKG and WikiData.

Links	Annotators			Mutual agreement
	I	II	III	
grfr:Leverkusen owl:sameAs wd:Q2938.	✓	✓	✓	✓
grfr:Hirschberg owl:sameAs wd:Q32058833.	✗	✓	✗	✗
grfr:Hirschberg owl:sameAs wd:Q630383.	✓	✓	✓	✓
grfr:Hirschberg owl:sameAs wd:Q468337.	✓	✓	✗	✓
grfr:Weinheim owl:sameAs wd:Q7050.	✓	✓	✓	✓
grfr:Bielefeld owl:sameAs wd:Q2112.	✓	✓	✓	✓
grfr:Osnabrück owl:sameAs wd:Q2916.	✓	✓	✓	✓
grfr:Osnabrück owl:sameAs wd:Q33158934.	✓	✓	✓	✓
grfr:Darmstadt owl:sameAs wd:Q2973.	✓	✓	✓	✓
grfr:Regensburg owl:sameAs wd:Q2978.	✓	✓	✓	✓
grfr:Regensburg owl:sameAs wd:Q2978.	✓	✓	✓	✓
grfr:Unna owl:sameAs wd:Q3949.	✓	✓	✓	✓
grfr:Olching owl:sameAs wd:Q32206345.	✓	✓	✓	✓
grfr:Olching owl:sameAs wd:Q178486.	✓	✓	✓	✓
grfr:Essen owl:sameAs wd:Q2066.	✓	✓	✓	✓
grfr:Hockenheim owl:sameAs wd:Q32059421.	✓	✓	✗	✓
grfr:Dresden owl:sameAs wd:Q1731.	✓	✓	✓	✓
grfr:Oberhausen owl:sameAs wd:Q32200661.	✓	✓	✓	✓
grfr:Oberhausen owl:sameAs wd:Q32200649.	✓	✓	✓	✓
grfr:Neuss owl:sameAs wd:Q2948.	✓	✓	✓	✓
grfr:Leutershausen owl:sameAs wd:Q389945.	✓	✓	✓	✓
grfr:Heidelberg owl:sameAs wd:Q2966.	✓	✓	✓	✓
grfr:Leverkusen owl: sameAs wd:Q2938.	✓	✓	✓	✓
grfr:Schwerte owl:sameAs wd:Q6863.	✓	✓	✓	✓
grfr:Wolfratshausen owl:sameAs wd:Q503160.	✓	✓	✓	✓
grfr:Hemsbach owl:sameAs wd:Q81012.	✓	✓	✓	✓
grfr:Werdohl owl:sameAs wd:Q5575.	✓	✓	✓	✓
grfr:Ketsch owl:sameAs wd:Q32064800.	✓	✓	✓	✓
grfr:Dossenheim owl:sameAs wd:Q31971934.	✓	✓	✓	✓
grfr:Bremen owl:sameAs wd:Q1209.	✓	✓	✗	✓
grfr:Bremen owl:sameAs wd:Q24879.	✓	✓	✓	✓
grfr:Paderborn owl:sameAs wd:Q2971.	✓	✓	✓	✓
grfr:Bobenheim-Roxheim owl:sameAs wd:Q31916736.	✓	✓	✓	✓
grfr:Mannheim owl:sameAs wd:Q2119.	✓	✓	✓	✓

## Usage Notes

Representing the annotated graffiti images as an RDF knowledge graph promises to facilitate many applications and use cases. In the project web site (https://dice-group.github.io/ingrid.github.io/), we provide some commonly asked SPARQL query examples that demonstrate many real world use cases. We outline some of which within this section.

### Data retrieval

**Fig. 9:**
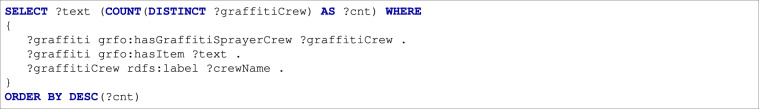
How many crews painted each graffiti?

**Fig. 10:**
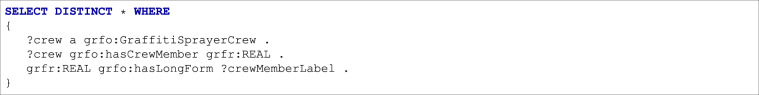
In which crews does the crew member “REAL” work?

While our database of the Ingrid project contains a significant number images, they are not represented in a format optimized for retrieval. By providing IngridKG in RDF with a well-defined ontology, we enable the easy retrieval of data with a structured query languages such as SPARQL. For example, in Fig. 9 we show a query to retrieve an ordered list of number of crews participated in painted each graffiti. Another query to retrieve all crews where a specific crew member works is provided in Fig. 10.

### Geographic queries

**Fig. 11:**
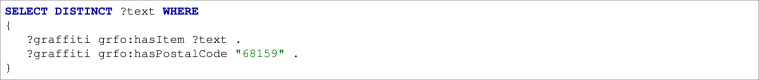
List all graffiti text on the “68159” postal code.

Modelling the geographic information associated with each graffiti in our knowledge graph adds a considerable amount of value when to aggregate different pieces of information based on geographic location. For example, Fig. 11 shows a SPARQL query to list all graffiti text for all graffiti within the area of the postal code “68159”.

### Textual data analysis

**Fig. 12:**
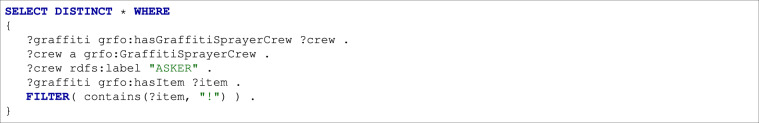
Find all graffiti drawn by a crew named “ASKER” and item field contains “!”.

Our knowledge graph allows to obtain a deeper analysis of textual data within annotation. For example, Fig. 12 shows a SPARQL query to find all graffiti drawn by a crew named “ASKER” and item field contains “!”.

### Computing statistics

**Fig. 13:**
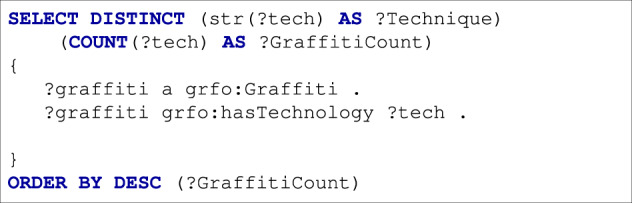
Retrieve the count of graffiti per individual technique.

The reach annotations of the graffiti within IngridKG enable the easy computation of statistical aspects of the data. For example, Fig. 13a, we show a SPARQL query that counts the number of graffiti drawn per drawing technique. In Fig. 13b, we show the resulted statistics using the last version of our knowledge graph.

## Data Availability

Due to different contractual conditions, only a subset of the photographs of the graffiti are public, with those provided by police departments in Mannheim, Munich and Cologne are not publicly visible. This does not apply to the metadata of the photographs which were compiled by our annotators, all of which are public. All the photographs provided by the private collector *Peter Kreuzer* together with its annotations are publicly available. Note that, some parts of the publicly available graffiti’s photographs are distorted for data protection reasons. For data protection reasons, the annotators’ names are not publicly available. All the data within IngridKG is publicly available under the *Creative Commons Attribution 4.0 International* license. All our resources are served from one of our servers via **persistent URIs**. For an example resource see (grfr:64681grfr:64681). The resource is maintained by the DICE research team (https://dice-research.org) as part of the lab’s Hobbit dataset efforts^6^. A 100TB-Server maintained by the Paderborn university’s computing center is hosting the knowledge graph. We also provide **dump files** of our dataset for download. IngridKG dump files are located on our Hobbit storage https://hobbitdata.informatik.uni-leipzig.de/INGRID/ and **archived on** ***Zenodo***^3^. Finally, we publicly serve IngridKG via a **SPARQL endpoint** (https://graffiti.data.dice-research.org/sparql). Table 3 summarizes all technical details of our knowledge graph pertaining to its availability.

## Code availability

Our source code to generate the new versions of IngridKG is publicly available at (https://github.com/dice-group/Ingrid) and will be maintained in parallel with the knowledge graph. We provide our source code under the software license of GPL 3.0 (https://www.gnu.org/licenses/gpl-3.0).

## References

[1] Ngomo, A.-C. N., Auer, S., Lehmann, J. & Zaveri, A. Introduction to linked data and its lifecycle on the web. In *Reasoning Web International Summer School*, 1–99 (Springer, 2014).

[2] Wilkinson, M. D. *et al*. The fair guiding principles for scientific data management and stewardship. *Scientific data* **3** (2016). 10.1038/sdata.2016.18 PMC4792175 26978244

[3] Sherif MA, da Silva AAM, Pestryakova S, Ahmed AF, Ngomo A-CN (2023). Zenodo.

[4] Ngonga Ngomo, A.-C. *et al*. LIMES - A Framework for Link Discovery on the Semantic Web. *KI - Künstliche Intelligenz, German Journal of Artificial Intelligence - Organ des Fachbereichs “Künstliche Intelligenz” der Gesellschaft für Informatik e.V*. (2021).

[5] Jain, T., Lennan, C., John, Z. & Tran, D. *Imagededup.* https://github.com/idealo/imagededup (2019).

[6] Röder, M., Kuchelev, D. & Ngonga Ngomo, A.-C. Hobbit: A platform for benchmarking big linked data. *Data Science* 1–21 (2019).

